# Assessment of serum tumor markers CEA, CA-125, and CA19-9 as adjuncts in non-small cell lung cancer management

**DOI:** 10.18632/oncotarget.28566

**Published:** 2024-06-13

**Authors:** Scott Strum, Mark Vincent, Meghan Gipson, Eric McArthur, Daniel Breadner

**Affiliations:** ^1^Department of Oncology, Schulich School of Medicine and Dentistry, London, ON, Canada; ^2^London Regional Cancer Program at London Health Sciences Centre, London, ON, Canada; ^3^Department of Medicine, Royal College of Surgeons in Ireland, Dublin, Ireland

**Keywords:** tumor marker, biomarker, lung cancer, NSCLC, translational research

## Abstract

Conventional tumor markers may serve as adjuncts in non-small cell lung cancer (NSCLC) management. This study analyzed whether three tumor markers (CEA, CA19-9, and CA-125) held associations with radiographic and clinical outcomes in NSCLC. It constituted a single-center study of NSCLC patients treated with systemic therapy at the London Regional Cancer Program. Serum tumor markers were analyzed for differences in radiographic responses (RECIST v1.1 or iRECIST), associations with clinical characteristics, and all-cause mortality. A total of 533 NSCLC patients were screened, of which 165 met inclusion criteria. A subset of 92 patients had paired tumor markers and radiographic scans. From the latter population, median (IQR) fold-change from nadir to progression was 2.13 (IQR 1.24–3.02; *p* < 0.001) for CEA, 1.46 (IQR 1.13–2.18; *p* < 0.001) for CA19-9, and 1.53 (IQR 0.96–2.12; *p* < 0.001) for CA-125. Median (IQR) fold-change from baseline to radiographic response was 0.50 (IQR 0.27, 0.95; *p* < 0.001) for CEA, 1.08 (IQR 0.74, 1.61; *p* = 0.99) for CA19-9, and 0.47 (IQR 0.18, 1.26; *p* = 0.008) for CA-125. In conclusion, tumor markers are positioned to be used as adjunct tools in clinical decision making, especially for their associations with radiographic response (CEA/CA-125) or progression (CEA/CA-125/CA-19-9).

## INTRODUCTION

Lung cancer in Canada is one of the most common solid malignancies in North America. It holds a poor prognosis, with an average 5-year survival of less than 19% for all-comers [1]. Accurately diagnosing, prognosticating, monitoring, and treating lung cancer is crucial to lung cancer management. Currently, disease quantification and monitoring rely heavily on radiographic and clinical assessment, and there remain few widely used biochemical tools to assist in this evaluation. Newer technologies such as ctDNA have started to transform the landscape of minimally invasive techniques that can facilitate cancer screening and early diagnosis, enhance prediction and prognostication, correlate with staging, profile cancer-associated mutations and genomic alterations, and monitor for treatment response and resistance [2]. However, such technologies still have a number of hurdles to overcome before they are readily adopted into widespread clinical practice, of which cost is one [3, 4].

To date, a number of studies have explored the utility of readily available, low-cost tumour biomarkers in the assessment and management of NSCLC, spanning the breadth of screening [5], diagnosis and staging [6, 7], prognostication [8–19], prediction [13, 20–24], and monitoring/surveillance [13, 25–28]. However, most have been specific to a particular disease stage and/or treatment, or reported biomarker level measurements at only a single timepoint for their association with clinical or radiographic features. Furthermore, recent international NSCLC guidelines offer either no recommendations on the utility of conventional serum tumor markers in disease management, or do not suggest their use altogether [29–34]. However, the evidence for these recommendations is weak. Thus, there is clearly an unmet need for further exploration of their application in this disease space.

The aim of this retrospective study was to provide additional evidence for the clinical use of conventional serum tumor markers CEA, CA19-9, and CA-125 in NSCLC management. These markers have been analyzed in similar contexts as cited above, were reliably accessible at the index institution at the time of study, and are widely available in clinical settings for measurement. Results of this research are positioned to provide further support for their application in NSCLC.

## RESULTS

**Figure 1:**
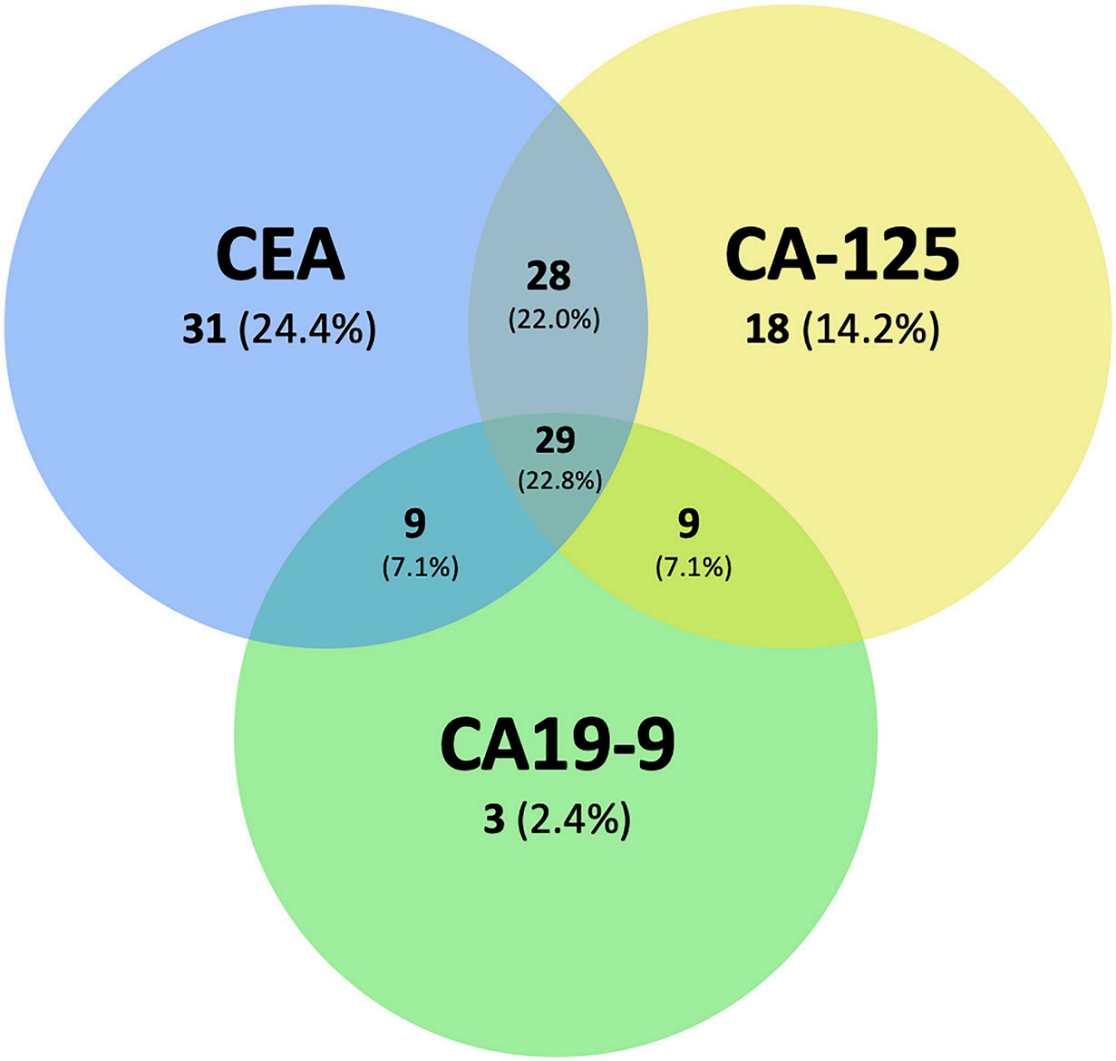
Venn diagram of the distribution of number of patients with elevated tumor markers who had at least one marker above the ULN at baseline (ULN) in Cohort A. Overlaps represent combinations of elevated tumor markers elevated above the ULN at baseline. Percentages represent the fraction of patients in each subgroup relative to those with at least one tumor marker elevated above the ULN at baseline.

A total of 533 NSCLC patients were identified for screening at London Health Sciences Centre, London, Ontario, Canada. 165 patients met inclusion criteria for Cohort A, and 92 patients for Cohort B. Patients were most commonly excluded due to failing to meet tumor marker and/or imaging investigation measurement within the timeframes detailed in the Methods section above. Baseline demographics between Cohort A and B were similar (Table 1). In cohort A, patients most commonly had stage IV disease (69.7%), adenocarcinoma histology (77.0%), and had a median age of 65 years. At baseline 58.8% of patients had an elevated CEA >ULN, 50.9% had CA-125 >ULN, and 30.3% had CA19-9 >ULN (Table 1). Figure 1 demonstrates the distribution of elevated tumor markers patients in Cohort A who had at least one marker above the ULN at baseline.

**Table 1:** Baseline demographics of patients in Cohort A (*n* = 165; baseline tumor markers only) and Cohort B (*n* = 92; paired tumor markers and radiographic scans)

	Cohort A	Cohort B		Cohort A	Cohort B		Cohort A	Cohort B
**Chemotherapy**			**Histologic subtype**			**Stage**		
Yes	55.2%	65.2%	Adenocarcinoma	77.0%	77.2%	I	1.2%	1.1%
No	43.6%	34.8%	Squamous cell	16.4%	17.2%	II	2.4%	2.2%
**Radiation**			Large Cell	0.6%	1.1%	III	24.2%	35.9%
Yes	38.8%	43.5%	Other/Missing	6.0%	4.4%	IV	69.7%	59.8%
No	60.0%	56.5%	**Sex**			**Elevated biomarker at baseline (>ULN)**		
**Immunotherapy**			Male	50.9%	46.7%	CEA	58.8%	54.3%
Yes	35.2%	46.7%	Female	49.1%	53.3%	CA-125	50.9%	46.7%
No	63.6%	56.5%	**Age**			CA19-9	30.3%	22.8%
**Surgery**			Median	66	64			
Yes	4.8%	3.3%						
No	93.9%	96.7%						

In Cohort A, demographic and clinical characteristics were analyzed for their association with elevated CEA at baseline. Adenocarcinoma was more likely to be associated with a CEA >ULN at baseline (Risk Ratio (RR) 1.36, 95% CI 1.12–1.66, *p* < 0.001), and squamous cell carcinoma was less likely (RR 0.29, 95% CI 0.14–0.63, *p* < 0.001). Stage III disease was less likely to be associated with CEA >ULN at baseline (RR 0.70, 95% CI 0.41–1.20, *p* < 0.001), while stage IV disease was more likely (RR 1.27, 95% CI 1.01–1.58, *p* < 0.001). Tumor EGFR or KRAS mutation statuses were not associated with an elevated CEA at baseline (EGFR RR 1.00, 95% CI 0.88–1.13, *p* = 0.98; KRAS RR 1.03, 95% CI 0.90–1.18, *p* = 0.63) (Table 2). In patients with stage IV disease in Cohort A, a survival analysis was completed to determine if there was a relationship with elevated CEA levels at baseline and all-cause mortality. A total of 69 patients (60.0%) of 115 died during follow up. An elevated baseline CEA was not associated with a difference in overall survival (HR 1.05, 95% CI 0.63–1.74, *p* = 0.84).

**Table 2:** Associations of demographic features with CEA level above the ULN at baseline for patients in Cohort A (*n* = 165) analyzed using risk rations

Variable	Value	Total *N*	Percent with CEA>5	Risk ratio	95% CI		*p*-value
**NSCLC subtype**	**Adenocarcinoma** yes	127	66.1%	**1.36**	1.12	1.66	<0.001
	**Adenocarcinoma** no	36	33.3%	*1.00 (referent)*			
	**SCC** yes	27	39.6%	**0.29**	0.14	0.63	<0.001
	**SCC** no	136	64.7%	*1.00 (referent)*			
**Stage**	**Stage III** yes	40	50.0%	**0.70**	1.41	1.20	<0.001
	**Stage III** no	125	61.6%	*1.00 (referent)*			
	**Stage IV** yes	115	64.3%	**1.27**	1.01	1.58	<0.001
	**Stage IV** no	50	46.0%	*1.00 (referent)*			
**Mutation**	**EGFR** yes	22	59.1%	**1.00**	0.88	1.13	0.98
	**EGFR** no	143	58.7%	*1.00 (referent)*			
	**KRAS** yes	27	63.0%	1.03	0.90	1.18	0.063
	**KRAS** no	138	58.0%	*1.00 (referent)*			

All values were reported with corresponding confidence intervals and *p*-value.

**Figure 2:**
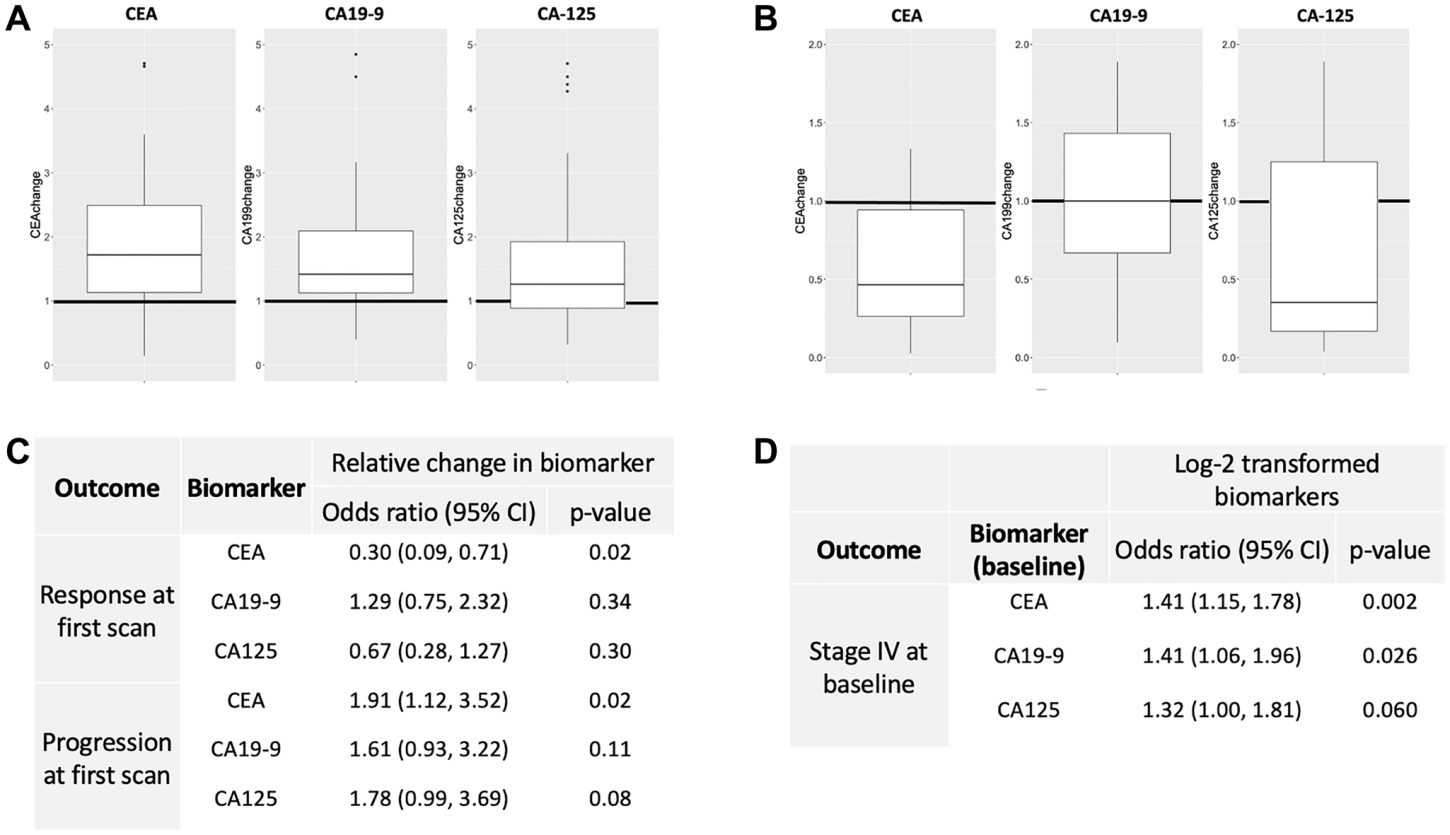
Patients from Cohort B were analyzed. (**A**) Box and whisker plots for the median fold-change of CEA, CA19-9, and CA-125 at radiographic progression relative to nadir. All demonstrated statistically significant differences. (**B**) Box and whisker plots for the media fold-change of CEA, CA19-9, and CA-125 at radiographic response relative to baseline. CEA and CA-125 demonstrated statistically significant differences. (**C**) Odds ratios for the relative change in each tumor marker level at first follow-up radiographic scan after systemic treatment was initiated. (**D**) Odds ratios for observing an elevation in biomarker levels at baseline in patients with stage IV disease.

Cohort B was analyzed for differences in tumor marker levels at response and progression relative to levels at baseline and nadir, respectively. Median (IQR) fold-change in tumor markers from nadir to progression were 2.13 (IQR 1.24–3.02; *p* < 0.001) for CEA (*n* = 47), 1.46 (IQR 1.13-2.18; *p* < 0.001) for CA19-9 (*n* = 46), and 1.53 (IQR 0.96–2.12; *p* < 0.001) for CA-125 (*n* = 47) (Figure 2A). Median (IQR) fold-change in tumor markers from baseline to their level at radiographic response (complete or partial) were 0.50 (IQR 0.27–0.95; *p* < 0.001) for CEA (*n* = 39), 1.08 (IQR 0.74–1.61; *p* = 0.99) for CA19-9 (*n* = 35), and 0.47 (IQR 0.18–1.26; *p* = 0.008) for CA-125 (*n* = 35) (Figure 2B). Thus, all three tumor markers showed significant increases at radiographic progression relative to nadir, and CEA and CA-125 showed significant decreases at radiographic response relative to baseline.

Cohort B was then analyzed for the relative change in tumor marker level at first follow-up radiographic scan after systemic treatment was initiated. Each 100% increase in CEA from baseline to first scan was associated with 70% lower odds of having a response at first scan (OR 0.30, 95% CI 0.09–0.71, *p* = 0.020) (Figure 2C). Similarly, each 100% increase in CEA from baseline to first scan was associated with 91% higher odds of progression (OR 1.91, 95% CI 1.12–3.52, *p* = 0.024) at first scan (Figure 2C). Similar associations were not seen for CA19-9 nor CA-125. The presence of stage IV disease did demonstrate significant OR with elevations in CEA and CA19-9 at baseline (Figure 2D). Lastly, using the tumor marker thresholds defined above in the Methods section, sensitivity and specificity for a >25% increase in CEA and radiographic progression were 47.5% and 80.0%, respectively. Sensitivity and specificity were 37.0% and 80%, respectively, for radiographic response.

## DISCUSSION

Lung cancer is one of the most common solid tumor diagnoses, and is often associated with a poor prognosis. One minimally invasive tool that can assist in the assessment of NSCLC are conventional tumor biomarkers, with prior studies reporting their utility in screening [5], diagnosis and staging [6, 7], prognostication [8–19], prediction [13, 20–24], and monitoring/surveillance [13, 25–28]. Despite this data, there still remains a lack of consensus around the role that these biomarkers play in disease management, and a paucity of information regarding disease monitoring while on treatment. To help strengthen the evidence base for the use of CEA, CA-125, and CA-19-9 in NSCLC management, this study offers further data to support their use in this setting.

In Cohort A, baseline biomarker levels were assessed for their association with a series of demographic and clinical features (Table 2). Elevated baseline CEA associated with a higher likelihood of stage IV disease and adenocarcinoma histology, the latter or which has been corroborated in at least one other study [35]. No associations with EGFR or KRAS mutation status and expression were found. This contrasted from a prior report [20], however it was restricted to stage IIIB and IV patients and assessed only the role of tyrosine kinase inhibitors in disease management. Lastly, elevated baseline CEA levels in patients with stage IV disease were not associated with overall survival. While this is supported by data from at least one publication [25], others have reported a worse prognosis with higher baseline CEA levels in advanced disease [8, 11, 14]. Thus, important trends are emerging, but unifying the data that already exists will help further contextualize and enhance the findings reported herein.

Importantly, in Cohort B, CEA, CA-125, and CA19-9 all demonstrated significant fold-increases at radiographic progression relative to nadir (Figure 2A). Similarly, CEA and CA-125 were significantly lower than baseline levels for those who responded radiographically (Figure 2B). Relative changes in CEA levels were associated with radiographic progression or response at first follow-up scan (Figure 2C), and the presence of stage IV disease at baseline did associate with elevated CEA and CA19-9 levels (Figure 2D). These important findings offer further support for their use as clinical adjuncts in disease management. Specific applications may include increasing the pre-test probability of disease progression/response on imaging scans when there is radiographic equipoise, or when monitoring for disease response/progression when access to imaging investigations is either delayed or unavailable. These results were found to be statistically significant in a patient population encompassing a wide cross-section of disease histologies, stages, and treatment types.

As with all studies, limitations in methodology and analysis did exist. First, patients selected for tumor marker measurement were not standardized. Ordering of serum CEA, CA-125, and CA19-9 was not standard of care at London Health Sciences Centre at the time of data collection, which could have led to selection bias. Additionally, patients were analyzed from all stages and histologies, but there was a significant over-representation of stage III and IV patients. Thus, applying the conclusions drawn to stage I and II disease may not be clinically appropriate, and should be interpreted with caution. And lastly, sensitivity and specificity using the pre-specified tumor marker change cutoffs were modest. A similar specificity of 70% has been reported in a meta-analysis of six trials studying outcomes of NSCLC patients while on treatment [36], but improved standardization of data collection and broader planned subgroup analyses would likely lead to enhanced sensitivity and specificity.

Notwithstanding the aforementioned limitations, between the pre-existing literature around conventional tumor markers in NSCLC and the results found in this study, there is ample evidence to suggest a need to further explore the clinical utility of these tools. One method would be through a prospective clinical trial, which allows for structured tumor marker data collection with a more homogeneous dataset, reducing bias and enhancing statistical validity. For example, in this study baseline imaging was permitted up to 60 days prior to therapy initiation, which could have led to the first on-treatment scan falsely identifying progression; a prospective study would allow for scheduled measurement of tumor marker levels that would limit this bias. It could also provide data about lead-times that would analyze for prediction of response/progression based on tumor marker changes, for which there exists preliminary supporting data from prior research [25, 27, 28, 37, 38]. Lastly, pre-specified subgroup analyses as well as the study of other biomarkers such as CYFRA21-1, NSE, and CA15-3 could also be completed. In addition to a prospective trial, with the wealth of information that exists in the literature, a meta-analysis would provide a high level of evidence to support the use of these conventional tumor markers in routine clinical care.

In conclusion, these inexpensive, widely available tests with rapid turnaround times and relatively short half-lives (CEA, CA-125, and CA19-9) are perfectly situated to serve as adjunctive clinical tools in the management of NSCLC. They are uniquely positioned to add value to patient care, especially in settings where ctDNA monitoring may not be available.

## MATERIALS AND METHODS

This study was a retrospective single-center review of patients treated at the London Regional Cancer Program in Ontario, Canada. Patients must have had a biopsy-proven diagnosis of NSCLC and received systemic treatment by a medical oncologist between January 1, 2016 and August 1, 2020. Patients must have been 18 years of age or older at the time of treatment, and were excluded if they had an active synchronous cancer, or a known concurrent non-cancerous disease that would have directly confounded results. Research Ethics Board approval was obtained before study initiation. All data were de-identified and stored in a secure REDCap database [39]. Demographic information collected for each patient included age, sex, disease stage at the time of treatment, histology, tumor genomic alterations, year and month of death (if known), as well as treatment modalities used during the relevant study window.

Patients included were divided into two cohorts. Cohort A comprised patients with radiographic imaging and tumor markers levels at baseline that were measured within 60 days prior to, or up to 14 days after, the initiation of systemic therapy. Cohort B was a subset of Cohort A, which included patients with paired radiographic imaging and tumor marker levels at both baseline and at least one follow-up time-point. At radiographic progression or response, tumor markers were included if they were drawn within 30 days of the associated scan. Disease progression or response was determined radiographically using RECIST 1.1 [40] or iRECIST [41] criteria. Tumor biomarker upper limits of normal (ULN) were defined as ≤5.0 Eμg/L for CEA; ≤35 U/mL for CA19-9; and ≤35 U/mL for CA-125.

For Cohort A, associations with demographic and clinical characteristics with elevated CEA levels at baseline were directly estimated. Association of elevated baseline CEA and all-cause mortality for those diagnosed at stage IV was assessed with a hazard ratio and its 95% confidence interval, obtained from Cox proportional hazards regression. All two-sided *p*-values < 0.05 were considered statistically significant. All analyses were performed using R version 4.1.1.

For statistical analyses involving Cohort B, continuous variables were presented using medians (interquartile range (IQR)), and categorical variables were presented using frequencies (percentage). Changes in biomarker levels for responders were analyzed relative to baseline. Changes in biomarker levels at progression were analyzed relative to levels at nadir. Levels at response and progression were compared to baseline/nadir using the Wilcoxon signed rank test, accounting for the paired nature of the data. Fold-changes in biomarkers between baseline and the time of first imaging were also evaluated against disease classification at the time of first imaging. The association between response/progression and biomarker change at the time of first imaging was assessed using logistic regression to obtain odds ratios and their associated 95% confidence intervals. Lastly, the sensitivity and specificity of CEA for at radiographic progression and response were calculated. A threshold for change in CEA was set at a >25% increase from nadir any time prior to progression, and a >25% decrease from baseline to the time of response was set as the threshold for radiographic response. Thresholds were extrapolated from prior studies, as no standards are yet universally defined [13, 26, 28, 37, 38].
